# Preterm birth: the role of knowledge transfer and exchange

**DOI:** 10.1186/s12961-017-0238-0

**Published:** 2017-09-06

**Authors:** Hacsi Horvath, Claire D. Brindis, E. Michael Reyes, Gavin Yamey, Linda Franck, the Knowledge Transfer and Exchange (KTE) Working Group, Meg Autry, Carol Dawson-Rose, Donna DeRoo, Larissa Duncan, Carlina Hansen, Jenee Johnson, Mary Lynch, Sarah Macfarlane, Janet Myers, John Colin Partridge, Laura Schmidt, Diana Taylor

**Affiliations:** 1 Department of Epidemiology and Biostatistics, School of Medicine, University of California, San Francisco, 550 Sixteenth Street, third floor, San Francisco, CA 94158 United States of America; 2 Philip R. Lee Institute for Health Policy Studies, University of California, San Francisco, San Francisco, CA United States of America; 3 Global Health Sciences, University of California, San Francisco, San Francisco, CA United States of America; 4 Department of Pediatrics, School of Medicine, University of California, San Francisco, San Francisco, CA United States of America; 5 Department of Family and Community Medicine, School of Medicine, University of California, San Francisco, San Francisco, CA United States of America; 6 Present affiliation: Duke Global Health Institute, Duke University, Durham, NC United States of America; 7 Department of Family Health Care Nursing, School of Nursing, University of California, San Francisco, San Francisco, CA United States of America

**Keywords:** Preterm birth, Prematurity, Knowledge transfer, Knowledge transfer and exchange, Knowledge translation, Implementation science

## Abstract

**Background:**

Preterm birth (PTB) is the leading cause of death in children under age five. Healthcare policy and other decision-making relevant to PTB may rely on obsolete, incomplete or inapplicable research evidence, leading to worsened outcomes. Appropriate knowledge transfer and exchange (KTE) strategies are an important component of efforts to reduce the global PTB burden. We sought to develop a ‘landscape’ analysis of KTE strategies currently used in PTB and related contexts, and to make recommendations for optimising programmatic implementation and for future research.

**Methods:**

In the University of California, San Francisco’s Preterm Birth Initiative, we convened a multidisciplinary working group and examined KTE frameworks. After selecting a widely-used, adaptable, theoretically-strong framework we reviewed the literature to identify evidence-based KTE strategies. We analysed KTE approaches focusing on key PTB stakeholders (individuals, families and communities, healthcare providers and policymakers). Guided by the framework, we articulated KTE approaches that would likely improve PTB outcomes. We further applied the KTE framework in developing recommendations.

**Results:**

We selected the Linking Research to Action framework. Searches identified 19 systematic reviews, including two ‘reviews of reviews’. Twelve reviews provided evidence for KTE strategies in the context of maternal, neonatal and child health, though not PTB specifically; seven reviews provided ‘cross-cutting’ evidence that could likely be generalised to PTB contexts.

For individuals, families and communities, potentially effective KTE strategies include community-based approaches, ‘decision aids’, regular discussions with providers and other strategies. For providers, KTE outcomes may be improved through local opinion leaders, electronic reminders, multifaceted strategies and other approaches. Policy decisions relevant to PTB may best be informed through the use of evidence briefs, deliberative dialogues, the SUPPORT tools for evidence-informed policymaking and other strategies.

Our recommendations for research addressed knowledge gaps in regard to partner engagement, applicability and context, implementation strategy research, monitoring and evaluation, and infrastructure for sustainable KTE efforts.

**Conclusions:**

Evidence-based KTE, using strategies appropriate to each stakeholder group, is essential to any effort to improve health at the population level. PTB stakeholders should be fully engaged in KTE and programme planning from its earliest stages, and ideally before planning begins.

**Electronic supplementary material:**

The online version of this article (doi:10.1186/s12961-017-0238-0) contains supplementary material, which is available to authorized users.

## Background

Of more than six million deaths in children under the age of 5 in 2013, over one million died due to complications of preterm birth (PTB) [1], with PTB now being the leading cause of death in children under five [1]. The rate of decline in deaths from PTB has been much slower than the rate of decline in overall child deaths. While the all-cause under-5 mortality rate fell sharply from 2000 to 2013 from about 77 to 46 deaths per 1000 live births – an annual average rate of reduction of 4.1% per year – the annual average rate of reduction for deaths from prematurity was only 2.1% per year over the same time-period [1]. The causes of and solutions to PTB are complex and multifactorial.

Several important global efforts are currently in progress to address the large global burden of PTB. In 2014, the University of California, San Francisco (UCSF) launched the Preterm Birth Initiative [2]. The Preterm Birth Initiative is working initially in three locations in California (San Francisco, Alameda and Fresno counties) and three countries in east Africa (Kenya, Rwanda and Uganda) [2].

In August 2014, the Preterm Birth Initiative convened three distinct multidisciplinary working groups to respectively examine and summarise the evidence in three different landscapes, namely (1) the current landscape of PTB discovery science (i.e. research on causation and new pathways for prevention and treatment); (2) clinical and programmatic interventions for reducing the burden of PTB; and (3) strategies for improving ‘knowledge transfer and exchange’ (KTE) related to PTB. This paper was written by members of the KTE Working Group, which is devoted to the third of the three focus areas. KTE Working Group members had backgrounds in adolescent and women’s health, community-based service provision, clinical epidemiology, global health, health policy, HIV prevention, neonatology, nursing, obstetrics and gynaecology, and public health.

In this paper, we describe (1) our adoption of a conceptual ‘framework’ within which to articulate KTE strategies to improve the uptake and use of the best and most applicable research evidence – knowledge – by all PTB stakeholders; (2) present our review of the evidence for these strategies; and (3) provide recommendations for applying and improving these strategies when implementing programmes to reduce the burden of prematurity. Among other constituents, PTB stakeholders may include individual mothers and their infants, fathers and other family members, doctors, nurses and other healthcare providers, people who work in community-based organisations, researchers in PTB etiologies, prevention and care, and policymakers at levels from local to global.

### KTE

Over several decades, the concept of knowledge transfer (KT) has emerged to optimise the transfer of the latest research evidence and stakeholder perspectives, with the goal of improving health outcomes [3]. KT is the “*synthesis, exchange, and application of knowledge by relevant stakeholders to accelerate the benefits of global and local innovation in strengthening health systems and improving people’s health*” [4]. The scientific literature includes more than 100 different terms to characterise KT [5]. Although in a strict sense there are conceptual differences between two of the most commonly used terms, ‘knowledge transfer’ and ‘knowledge translation’, these terms may also be used synonymously [3, 5]. Cognizant of the role of ‘exchange’ in effective KT (as communication and exchange are vitally needed to ensure that research evidence is directly applicable to the needs and uses of several kinds of PTB stakeholders), we use the term KTE.

### Evidence-based medicine and PTB

Interventions to improve perinatal outcomes, including to mitigate the adverse sequelae of PTB, have figured large in the history of evidence-based medicine. The Oxford Database of Perinatal Trials [6], developed by Cochrane Collaboration co-founder Iain Chalmers, was a direct precursor to the Cochrane Central Register of Controlled Trials [7]. A 2005 paper by Hanney et al. [8] nicely summarises the history of research evidence on the use of antenatal corticosteroids for preventing respiratory distress syndrome in preterm neonates. The authors highlight the first randomised controlled trial of this intervention [9]. Liggins and Howie found “*sufficient evidence of beneficial effects on lung function and of absence of adverse effects to justify further trials*” [9] of antenatal corticosteroids [8]. Following additional trials of the intervention, Crowley [10] first synthesised the evidence in a systematic review and meta-analysis that was integral to the Cochrane Collaboration’s development in the early 1990s [8]. Despite the intervention’s uptake remaining suboptimal for many years in the United Kingdom, United States and other settings [8], it has undoubtedly saved many thousands of infant lives.

### Deploying community voices in advancing health agendas

Health research evidence is crucial to deploy in reducing PTB-related death and suffering, but it is only part of a continuum of knowledge that includes rich qualitative consumer and provider experience and very specific socioeconomic, structural and cultural considerations. Knowledge developed from health research evidence is valuable only to the extent that it is actually applicable to the lives and relevant to the specific concerns of those stakeholders by whom the evidence is meant to be used. While available systematic reviews may show high quality evidence for an intervention’s efficacy, this evidence may be only one of several factors that stakeholders take into account in healthcare decision-making [4].

### Barriers to implementation of evidence

Even when it is applicable, new knowledge developed from health research evidence is valuable only to the extent that it is actually used by stakeholders in healthcare. Healthcare providers are often unaware of the latest research and its implications for their own practice. As a result, individual patients often do not receive the best treatment or may receive unnecessary (and even harmful) treatments. Traditionally, patients may not be actively engaged in making decisions regarding their own healthcare options, may have been marginalised due to traditional roles and expectations in healthcare and, as a result, may often have played passive roles in seeking healthcare information. Healthcare costs are high because evidence on more cost-effective or more efficient delivery approaches is often unavailable or is ignored, or because ‘perverse incentives’ lead to unintended or undesirable outcomes (e.g. improved antenatal care leading to higher rates of caesarean section) [11]. Researchers needlessly investigate questions for which there is already high-quality evidence [12]. National and local policymakers face many barriers in the use of the latest research evidence as they make important resource allocation decisions [13], and their prioritisations may be based on obsolete or incomplete evidence, or even on ‘expert opinion’ alone.

As in most areas of healthcare, uptake and use of an evidence-based PTB intervention may depend its applicability to the lives of individuals and their families in a given setting. It is not just the intervention and how people assess the balance of its risks and benefits, but also the feasibility of its implementation, financial cost and qualitative factors such as cultural acceptance and perceived benefit within the community. The intervention’s primary outcomes (e.g. the survival of a very preterm infant in a very low-resource setting) may agree or conflict with community values and preferences and may carry significant ethical implications. Even when providers are aware of research evidence, and would be keen to use it, they may face financial, infrastructural, sociocultural or other barriers to incorporating it in their practice [3, 14]. Policymakers may lack appropriate skills for finding and using research evidence and assessing its local applicability, as well for framing policy questions and prioritising actionable recommendations based on available financial and community resources [13].

The net impacts of failure to account for local contexts, needs and perspectives may waste financial and human resources allocated to healthcare. Such failure also contributes to health policy recommendations that are poorly implemented or not implemented at all, or interventions that are not effective in specific settings despite evidence for efficacy. This can result in population-level health outcome indicators that do not improve [15].

### A knowledge ecosystem, founded on humility

**Fig. 1:**
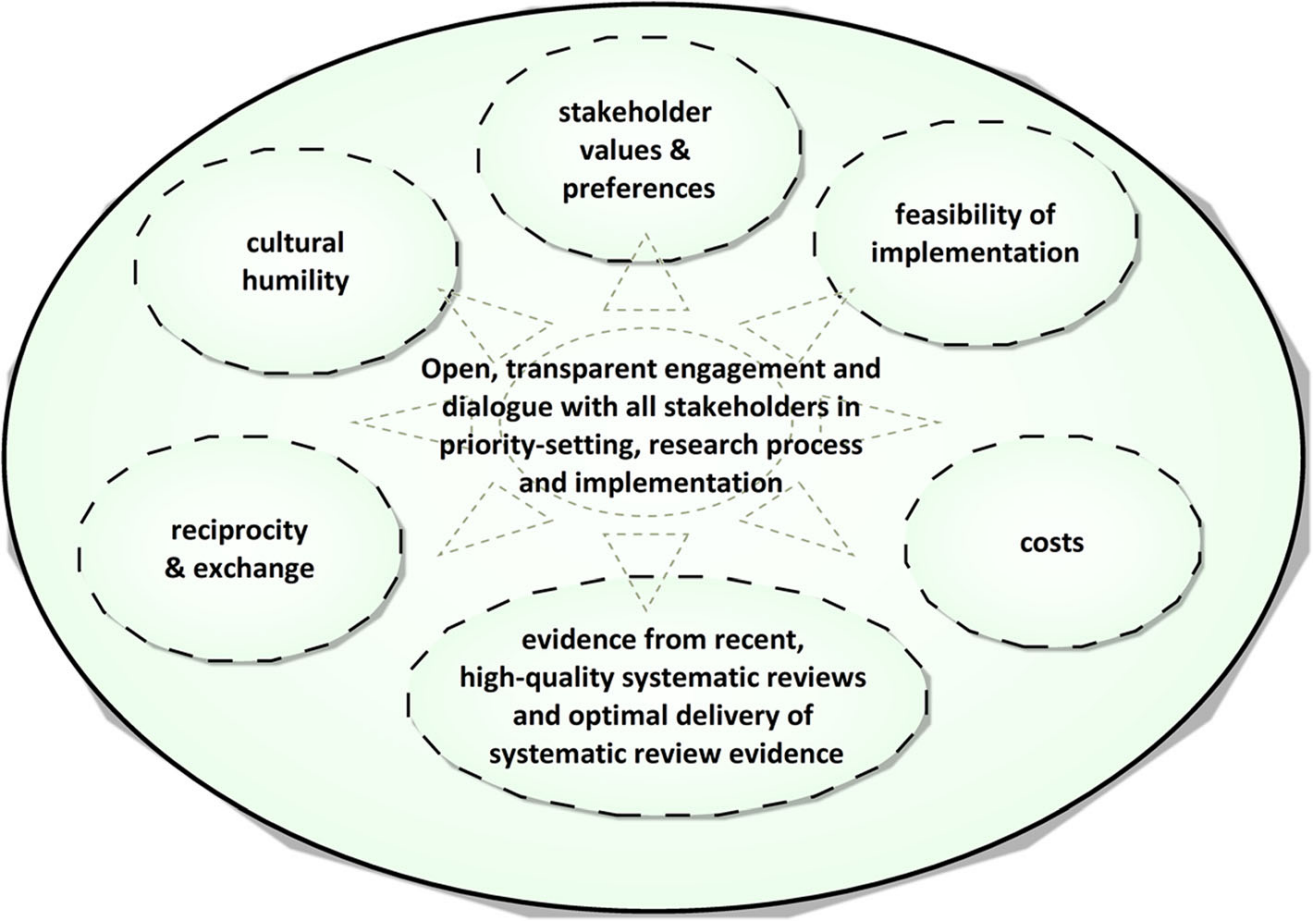
A knowledge transfer and exchange ‘ecosystem’ to improve the use of evidence in preterm birth

There are several effective strategies to improve the use of research evidence [3]. Knowledge of research evidence can indeed be communicated or ‘transferred’ from one stakeholder to another, but true communication thrives in reciprocity and exchange. By ‘exchange’ among stakeholders, we mean to suggest a healthy and equitable ‘ecosystem’ of health knowledge in which, at an early stage and on an ongoing basis, authentic voices from each constituency are given the opportunity to participate in research priority setting and the holistic entirety of the knowledge generation, implementation and evaluation process (Fig. 1). Such an equitable ecosystem involves an integrated and collaborative approach to decision-making in which researchers and implementers take seriously the knowledge and perspectives of all these stakeholders, and in which the means of communicating about health research evidence is optimised for each kind of audience.

As reciprocity enhances communication, so true exchange flourishes when its grounding principles aim for equity and inclusion among all concerned. By ‘equity and inclusion’, we mean to suggest that ‘real life’ relevance and applicability of new research and implemented programmes will increase to the extent that the values, preferences and ethical concerns of specific and local constituents are ‘heard’ within the knowledge ecosystem. Constituents’ views should not only be heard but also given appropriate consideration and, when feasible, integrated holistically into intervention planning and implementation. People whose knowledge, societal contexts and life experiences are honoured in this way will be more inclined to make use of health research evidence, and to consider this evidence in their decision-making.

Reflection on humility would not be wasted time for academics, researchers and other professionals hoping to helicopter into a clinic, ministry office or community centre with the latest systematic review in hand, intent on bringing this evidence to bear in healthcare decision-making. This is especially true in situations where the ‘target’ population has been systematically and institutionally targeted for disempowerment and violence over the course of many generations. Similarly, heightened cultural awareness is needed when the population has otherwise been deprived of its voice and presence in decision-making through being on the lower rungs of socioeconomic, political or cultural hierarchies. Because most world cultures are male-centred, including in the United States, women in these populations typically have had much less influence and agency in healthcare decision-making than their male counterparts. This is true in spite of the tremendously rich traditions of body-knowledge, pregnancy, childbirth and infant care that women in all settings have often shared with their peers, sisters, daughters, nieces, granddaughters and other women while still being nearly always responsible for primary care in their homes and families.

In many settings, racism and socioeconomic prejudice among individual physicians or nurses, whether conscious or unconscious, also may distort appropriate clinical practice and patient communication. This pervasive short-circuit among stakeholders often leads community members and whole communities to distrust the agendas of ‘healthcare’ and ‘health research’, particularly among those whose families and forebears have experienced multi-generational disempowerment and poverty.

### Approaches for framing KTE

KTE strategies are guided by intellectual constructs often described as ‘frameworks’. These KTE frameworks provide an architecture and rationale for the use of KTE strategies. Many KTE frameworks have been proposed in healthcare and other fields, but most have not been tested empirically [4]. KTE frameworks do not necessarily ‘conflict’ with each other; they can often provide complementary perspectives. In considering an equitable and inclusive knowledge ecosystem, it becomes clear that a KTE framework that prioritises open communication, collaboration and humility must be integral to all KTE and implementation efforts and, ultimately, healthcare decision-making. The KTE Working Group wished to identify such a model.

In addition to reviewing the scientific literature, the KTE Working Group consulted with a leading KTE expert (Dr John Lavis, a clinical epidemiologist who holds the Canada Research Chair in Evidence-Informed Health Systems; see http://fhs.mcmaster.ca/ceb/faculty_member_lavis.htm) and with local community-based organisations serving pregnant women and mothers at high-risk of PTB.

## Methods

### KTE framework

In July 2014, before the KTE Working Group was formally convened, three authors (GY, LF and HH) met in teleconference with Dr Lavis. Through this meeting, the authors identified several KT and KTE frameworks currently in use and achieved a more nuanced understanding of KTE goals in regard to engagement with different stakeholder groups. Dr Lavis subsequently visited UCSF and presented on KTE to our working group and other colleagues in the Preterm Birth Initiative.

The KTE Working Group also examined analyses of a range of important KT and KTE frameworks, including Linking Research to Action (Linking RTA), the Knowledge to Action Process, Promoting Action on Research Implementation in Health Services and other frameworks [3, 4, 16–21]. Our framing was helped by a 2012 analysis by the WHO Department of Ageing and Life-Course [4], in which a WHO-convened working group examined nine important frameworks and chose one to guide that department’s knowledge transfer activities.

### KTE strategies

We conducted a non-systematic ‘narrative’ review of the scientific literature in an effort to identify systematic and narrative reviews of efficacious KTE strategies, and ideally ‘systematic reviews of systematic reviews’ describing such strategies. The term ‘interventions’ may be appropriate when referring to KTE efforts, and indeed these efforts may themselves constitute health-related interventions. In this report, however, we use the term ‘strategies’ instead of ‘interventions’ when referring to KTE in order to mitigate potential confusion between clinical or programmatic interventions and KTE.

To facilitate analysis, we primarily discuss KTE in the context of three main stakeholder groups affected by PTB, namely (1) individuals and families, (2) healthcare providers and (3) policymakers. We consider KTE to refer to those strategies needed for the effective transfer and exchange of health-related research, scientific knowledge and lived human experience amongst all stakeholders in PTB.

## Results

### Results: KTE framework

The KTE Working Group conducted a comprehensive review and comparison of the major KT and KTE frameworks [3, 4, 15–21], applying such criteria as framework adaptability and utility in facilitating KTE with all important stakeholders of research relevant to PTB. We determined that the Linking RTA framework [17] would be the most valuable in organising our analyses.

Table 1 synthesises the Linking RTA framework, expanded with the KTE Working Group’s additional considerations.

**Table 1:** Linking RTA framework (adapted from Lavis et al. [16] and Grimshaw et al. [3])

Four key approaches for linking research to action	Four clusters of activities	Five questions
Helping to develop a culture in the target audience that values the use of research evidence; producing highly-relevant research evidence; transferring and exchanging knowledge in appropriate ways; and evaluating efforts for linking research to action.	‘Push’ efforts: These may be appropriate when knowledge users (e.g. individuals and families) do not realise that they should consider a particular health-related message, or perhaps intentionally disregard the message; examples of ‘push’ efforts could be a strategy using mass media, billboards, advertising etc. ‘User pull’ efforts: Many kinds of stakeholders actively seek healthcare information about particular urgent issues; an example of a ‘user pull’ effort would be KTE strategies that package high quality, highly relevant research evidence for each type of audience, distilling key findings of a systematic review to one or two pages. ‘Exchange’ efforts: Exchange efforts establish common ground in developing and prioritising research questions, deciding how to answer these questions and sharing other kinds of insights; an example of an ‘exchange’ effort would be ‘deliberative dialogues’, shared discussions in support of a decision-making process between a university research unit and a community-based organisation. ‘Integrated’ efforts: KTE strategies using large online KTE ‘platforms’, essentially ‘one-stop shops’ for health research evidence, can include elements of each approach described above.	1) What should be transferred? (i.e. what are the key messages?)2) To whom should research knowledge be transferred? (i.e. who is the audience?)3) By whom should research knowledge be transferred? (i.e. who is the messenger?)4) How should research knowledge be transferred? (i.e. with what communicative processes and infrastructure should the message be delivered?)5) With what effect should research knowledge be transferred and exchanged? (i.e. how may we evaluate the impact, including the impact on health equity, of KTE strategies? Have community voices really informed policy, practice and the research agenda?)

Note: Lavis et al. [16] suggest that evidence from systematic reviews should be the core of ‘what’ is transferred. Depending on the audience, the key messages arising from this evidence may vary and, therefore, may need to be packaged or presented differently. Lavis et al. [16] propose that answers to the remaining questions will also vary, depending on the context and setting

To assure fidelity, some text in this table is presented verbatim from the original sources

### Results: KTE strategies for PTB

An additional file (Additional file 1) describes our methods used in searching the literature and screening search results. We identified 19 reviews [3, 14, 16–18, 22–35], two of which [3, 14] were reviews of reviews. These fell generally into two categories, namely (1) reviews of KTE strategies irrespective of the healthcare intervention context in which they had been applied (‘Cross-cutting KTE’), and (2) reviews of KTE strategies applied specifically to maternal, neonatal and child health (MNCH) interventions (‘MNCH KTE’). Cross-cutting KTE reviews [3, 16–18, 22–24] considered the efficacy of specific KTE strategies in any area of healthcare. No MNCH KTE or cross-cutting KTE review directly addressed KTE strategies for improving outcomes relevant to PTB.

One particular cross-cutting ‘review of reviews’ provided an enhanced understanding of ‘what works’ in KTE strategies across all healthcare interventions. In addition to describing key concepts in KTE, this work by Grimshaw et al. [3] provided an excellent summary overview of cross-cutting KTE strategies. Grimshaw et al. [3] examined Cochrane reviews of KTE strategies for healthcare providers as well as healthcare ‘consumers’, i.e. individuals and their families. Many of these reviews are very large, including as many as 140 randomised controlled trials [23]. In examining the evidence for KTE strategies addressing individuals and providers, Grimshaw et al. [3] only considered Cochrane reviews. However, given the variable methodological quality of non-Cochrane reviews and the consistently high quality of Cochrane reviews [36–39], the working group judged this to be an appropriate resource. In the same paper [3], Grimshaw et al. also analysed the Supporting Policy-Relevant Reviews and Trials (SUPPORT) tools for facilitating KTE with policymakers [14].

### Key findings of the reviews

Overall, there was little systematic review evidence of direct relevance to KTE for preventing PTB or improving PTB outcomes. However, findings from MNCH KTE reviews and cross-cutting KTE reviews may be applicable to PTB stakeholders. ‘What works’ in each stakeholder group varies widely. Reviews of randomised trials focused on individual and on provider populations found significantly favourable results for many KTE strategies, often measured objectively. However, the evidence base for KTE strategies focused on policymakers is much more limited.

We did not formally assess evidence quality for KTE strategies (nor was this done in the other two reviews of reviews [3, 14]).

For individuals and families, many reviewed strategies resulted in improved MNCH outcomes. Efficacious strategies included ‘decision aids’ (informational tools to help guide decision-making) [3, 35], letting pregnant women carry their own case notes [34], regular discussions between providers and pregnant women [30], evidence-based community-discussions [33], other community-based approaches [26–29, 32], interactive computer-based health communication [3], personalised risk communication [3], and patient-provider communication before consultations [3].

For providers, most KTE evidence came from cross-cutting reviews. Efficacious strategies included audit and feedback [3, 23, 24], in-service training [3, 25], educational meetings [3, 25], educational outreach [3, 25], local opinion leaders [3], tailored interventions [3], and computerised reminders [3]. Multi-faceted approaches can increase provider uptake of evidence from systematic reviews [40]. It is important to note that the term ‘providers’ can comprise people (e.g. generalist and specialist physicians and nurses, allied health workers, community health workers, midwives, relief workers, skilled birth attendants, social workers, allied health workers, informal caregivers) as well as healthcare systems and settings (e.g. clinics, community centres, homes, hospitals and pharmacies).

As mentioned above, KTE evidence for strategies to change decision-making behaviour in health policymakers is limited, with the only outcome being the intention to use systematic review evidence in policymaking. Strategies reviewed included evidence briefs and deliberative dialogues based on these evidence briefs [22], online ‘one-stop shop’ of optimally-packaged key data from systematic reviews and other documents [17], ‘rapid response units’ to provide rapid guidance about best evidence [12], and the SUPPORT tools for evidence-informed policymaking [19].

Drawing on the Linking RTA framework, Tables 2, 3 and 4 provide a more detailed overview of key findings from all reviews. Findings are broken out by the stakeholder groups, including individuals and families (Table 2), healthcare providers (Table 3), and policymakers and other official stakeholders (Table 4).

**Table 2:** KTE with individuals, families and communities

Key barriers [14]:			
• Health education materials that do not address real situation, context, problems			
• Information provided passively, just for the sake of providing it, without active patient engagement			
• Insensitive attitude and behaviour of providers, power imbalance, lack of respect			
• Lack of translated materials and lack of qualified interpreters			
• Language and reading level (particularly for migrant populations and those with low literacy)			
• Low frequency of contact with provider			
Key facilitators [14]:			
• Care and support from family members, trained doulas and other kinds of care-giving beyond doctors and nurses			
• Continuous/frequent communication and exchange of information between healthcare providers and mothers rather than one-off contact or passive flow from provider			
• Group antenatal care, rather than one-to-one care			
• Continuity of care (particularly for maternal and newborn healthcare)			
• Integrated and comprehensive care (integrated care pathway model)			
Message	KTE strategy	Linking RTA approach	Outcomes
Healthy pregnancy; many other health topics [3, 35]	Decision aids (variety of approaches including leaflets, computer programmes, structured counselling, etc.)	Push or exchange (depending on modality)	Improved knowledge and accuracy in risk perception, improved active and informed decision-making. Reduced anxiety and improved ability to actually make decisions. No significant difference in birth outcomes (assessed in two small trials) in the context of decision aids related to breech presentation and to pain relief in labour.
			Greatest benefits were observed when a decision support technique was implemented in the form of counselling from a care provider involving information, discussion of options and clarification of values.
Healthy pregnancy [34]	Let pregnant women carry own case notes	User pull	Improved knowledge about own pregnancy and health.
Appropriate newborn care [30]	Regular discussions throughout pregnancy between providers and pregnant mothers	Exchange	Increased early initiation of breastfeeding.
Prevent child illness [33]	Information campaigns	Push	Improved immunisation uptake.
Prevent child illness [33]	Evidence-based community discussions	Exchange	Improved immunisation uptake.
Healthy pregnancy; appropriate newborn care [26–29, 31]	Community-based strategies to deliver information (e.g. use of community health workers, family-community service delivery, women’s groups)	Exchange	Depending on modality: Better prepared for birth; increased demand for information; increased use of antenatal clinics and delivery care; increased awareness about newborn care; decreased infant mortality; improved care-seeking for sick infants.
Improve knowledge, behaviour change (many topics) [3]	Interactive computer-based health communication applications	User pull	Improved knowledge, social support, clinical outcomes. Data were insufficient for meta-analysis of biological outcomes or analysis of cost effectiveness. Effects on these outcome categories remain unknown.
Understand risk, go for screening tests (many health topics) [3]	Personalised risk communication (written, spoken or visual)	Push	Uptake of screening tests. Low quality evidence, small effect size.
Improve engagement with patients (many topics) [3]	Communication before consultations (i.e. patient appointments with healthcare provider)	Exchange	Increased question-asking during consultations; increased patient participation in consultation; improved patient satisfaction. Both coaching and written material interventions produced similar effects on question asking, but coaching produced a larger increase in patient satisfaction. Overall, the benefits of ‘communication before consultations’ interventions were minor.

**Table 3:** KTE with providers

Key barriers [3, 14]:			
• Audit and feedback: challenges related to quality, sustainability and acceptance of audit, especially when enforced by an external agency			
• In-service training: neonatology: Need to reinforce good practices through adequate supervision, need for refresher courses, lack of standardised tools to evaluate the impact of training on health system goals, high costs, lack of capable trainers			
• Computerised reminders: less successful with more complex decision support systems, especially chronic disease management			
• Costs (especially with multifaceted interventions)			
• Structural and organisational capacity, shortages, long and irregular working hours, provider attitudes toward change (providers may resist change), provider competencies to build trust, comfort and patient centredness			
• Shortage of resources in health facilities			
• Variable standards of implementation of standard guidelines			
Key facilitators [3, 14]:			
• Audit and feedback: In general, larger effects were seen if baseline compliance was low			
• Educational meetings: Larger effects were associated with higher attendance rates, mixed interactive and didactic meetings and interactive meetings			
Message	KTE strategy	Linking RTA approach	Outcomes
Use most current evidence-based practice (neonatology and many other health topics) [3, 25]	In-service training and educational meetings; educational outreach	Exchange	Beneficial in improving provider compliance to standardised guidelines compared to receiving information leaflets and didactic lectures. Clearest and strongest effects with changing less-complex behaviours.
Use most current evidence-based practice (many health topics) [3]	Local opinion leaders	Exchange	Behaviour change, quality of care.
Use most current evidence-based practice (many health topics) [3, 23, 24]	Audit and feedback	Exchange	Behaviour change, quality of care.
Use most current evidence-based practice (many health topics) [3]	Tailored interventions	Depends on modality	Behaviour change, quality of care.
Use most current evidence-based practice (many health topics) [3]	Computerised reminders	Push or exchange (depending on modality)	Behaviour change, quality of care.
Use most current evidence-based practice (many health topics) [40]	Printed bulletin (mass-mailed to providers)	Push	“…*may improve evidence-based practice when there is a single clear message, if the change is relatively simple to accomplish, and there is a growing awareness by users of the evidence that a change in practice is required*.”
Use most current evidence-based practice (many health topics) [40]	Multifaceted interventions	Push or exchange (depending on modality)	Multifaceted interventions may be necessary to improve awareness and uptake of review evidence.

**Table 4:** KTE with policymakers

Key barriers [13]:			
• Lack of availability of evidence, lack of access to research and dissemination			
• Lack of clarity/relevance/reliability of research findings			
• Lack of timing/opportunity			
• Poor policymaker research skills			
• Costs (resource availability for evidence-based policy)			
Key facilitators [13]:			
• Availability and access to research/improved dissemination			
• Collaboration			
• Clarity/relevance/reliability of research findings			
• Relationship with policymakers			
• Relationship with researchers and those providing evidence			
‘Climate’ [17] and context of the health policy setting [43] have significant bearing on policymakers’ use of research evidence			
Message	KTE strategy	Linking RTA approach	Outcomes
Use updated systematic review evidence in developing health policy [22]	Evidence briefs	Facilitating user pull	Intention to use systematic review evidence.
Use updated systematic review evidence in developing health policy [22]	Deliberative dialogues based on evidence briefs	Exchange	Intention to use systematic review evidence.
Use updated systematic review evidence in developing health policy [18]	Systematic review-derived products: summaries of reviews, overviews of reviews and policy briefs	Facilitating user pull	Intention to use systematic review evidence.
Use updated systematic review evidence in developing health policy [17]	‘One-stop shop’ of optimally-packaged systematic review products and other key data, online	Integrated	Intention to use systematic review evidence.
Use updated systematic review evidence in developing health policy [17]	‘Rapid response units’ to provide written summaries, telephone consultations or in-person consultations about best evidence	Facilitating user pull	Intention to use systematic review evidence.
Use updated systematic review evidence in developing health policy [19]	SUPPORT tools for evidence-informed health policymaking	Depends	Intention to use systematic review evidence.

## Discussion

The landscaping process allowed the KTE working group to articulate an overview of strategies for improving uptake and use of ‘PTB knowledge’ by all stakeholders. The KTE working group could also then develop a research agenda specific to KTE.

### Priority activities for research, implementation and evaluation of KTE strategies

As the KTE Working Group assessed research priorities in broadly defined key stakeholder groups, it became clear that there were five cross-cutting thematic areas within which the research agenda could be articulated, helping to clarify the scope of research priorities. These thematic areas comprise ‘Partner engagement’, ‘Contextual research’, ‘KTE strategy research’, ‘Evaluation’ and ‘Infrastructure’ (Table 5).

**Table 5:** KTE research priorities for PTB

Thematic area	Individuals, families and communities	Providers	Policymakers	Across stakeholder groups
Partner engagement	Identifying and engaging with key informants and panel members	Engage with providers at each referral stage as well as in training	Understand ‘climate’ for use of research evidence	n/a
	Improve communication pathways with providers and policymakers	Learn more about how providers engage with affected lay populations and policymakers	Understand political, cultural, economic and other factors	
Contextual research	Understand what the most important PTB outcomes are in this population	Assess provider knowledge of efficacious interventions for preventing PTB and caring for mothers and infants affected by PTB	Know the degree to which PTB is prioritised in the health agenda	Learn how these stakeholders understand PTB
	Know their views on the optimal way forward for changing government and other health policies to affect those outcomes	Understand provider knowledge, skills and attitudes to implement and promote KTE strategies to reduce PTB Know what barriers exist to implementing KTE strategies	Understand barriers and facilitators making it a high priority in the specific setting	Learn about overall barriers and facilitators to stakeholder uptake of PTB knowledge
KTE strategy research	For example:• Decision aids• Let pregnant women carry own case notes• Regular and frequent discussions between providers and pregnant women/mothers• Information campaigns• Evidence-based community discussions• Community-based strategies• Interactive computer-based health communication apps• Personalised risk communication• Communication before consultations	For example:• In-service training and educational meetings; educational outreach• Local opinion leaders• Audit and feedback• Tailored interventions• Computerised reminders	For example:• Evidence briefs• Deliberative dialogues based on evidence briefs• Systematic review-derived products• ‘One-stop shop’ of optimally-packaged systematic review products and other key data, online• ‘Rapid response units’• SUPPORT tools for evidence-informed health policymaking	Investigate best ways to engage in KTE across all stakeholder groups, collaborating in their respective ways for KTE around PTB
Monitoring, learning and evaluation	*Pre- and post-intervention impact in terms of:*	*Pre- and post-intervention impact in terms of*:	*Pre- and post-intervention impact in terms of:*	*Pre- and post-intervention impact in terms of:*
	1) Access to systematic review evidence on PTB in community and from providers; 2) Changes in individual and community beliefs and norms in regard to cultural relevance and effectiveness; 3) Repeated data comparisons over the long-term; and 4) Overall changes in PTB outcomes	1) Access to systematic review evidence on PTB and KTE; 2) Adoption of systematic review evidence into new practice; 3) Repeated data comparisons over the long term; and 4) Overall changes in PTB outcomes	1) Access to systematic review evidence on PTB and KTE; 2) New policies informed by systematic review evidence; 3) Repeated comparisons of policies relevant to PTB and its determinants over the long term; and 4) Overall changes in PTB outcomes	Collaborative KTE efforts across stakeholder groups
Infrastructure	Science-based public education and outreach initiatives, especially with digital and social media platforms	PTB health evidence web portal for providers, with additional components for training and other services	‘One-stop shop’ on PTB for policymakers that would include optimally packaged online systematic review products and other key data, as well as rapid response units and other capacities	n/a

### Partner engagement

To develop long-term sustainable partnerships and networks for KTE research implementation, it is necessary to understand the diversity of stakeholders in KTE, how members of these constituencies interact with and affect one another, and how to achieve equitable transfer and exchange of findings. This task varies among stakeholder groups.

- For individuals, families and communities, KTE efforts should include identifying and engaging with key informants, as well as community organisations and other entities with capacity to promote KTE around PTB. Another goal would be to forge communication pathways between affected lay populations and researchers, the better to inform PTB research and implement prioritised programmes.
- For providers, KTE efforts should include engaging with all types of providers at each stage of referral, including during the course of their training, and learning more about the ways in which providers engage with other stakeholders.
- For policymakers, KTE efforts should include determining the ‘climate’ for use of research evidence in their given settings, and understanding the political, economic and cultural factors that influence policy development and implementation.

In a provocative but insightful paper, Cairney and Oliver [41] point out that evidence-based health policymaking is quite a different animal from the practice of evidence-based medicine. Especially at the national level, it is a complex and often unpredictable arena that functions within its own frames of reference and has its own bureaucracies, coded language and rules. Policymakers tend to operate within an ethos of ‘bounded rationality’, as they frequently feel they must make quick decisions without having had sufficient time to comprehend the evidence put before them. In order to make decisions, policymakers may use rational or irrational ‘short-cuts’ to gathering evidence. Their interest in the research evidence may wax and wane depending on external considerations. In view of this complicated environment, academics and others hoping to optimise uptake of research evidence in health policymaking at the national level should consider approaching the policymaking process and its requirements in completely different ways than they have done before. Cairney and Oliver [41] provide two key messages for academics and researchers (numbering added):

1. “[R]*ecognise the tendency of policymakers to base judgements on their well-established beliefs and shortcuts based on their emotions and familiarity with information. On that basis, consider how to reduce ambiguity, to persuade policymakers to frame a problem primarily in one particular way and, therefore, to demand scientific evidence to help solve that problem*.”
2. “[L]*earn ‘where the action is’, and be prepared to engage in a long-term strategy to be in a position to influence policy. In other words, identify ‘the action’ at several levels of government, learn the ‘rules of the game’ in institutions and networks (or the ‘venues’ in which policymaking takes place), form coalitions with like-minded actors, and work with the ‘policy entrepreneurs’ possessing the skills to exploit ‘windows of opportunity’ to give policymakers the motive and opportunity to adopt new solutions*.”

Cairney and Oliver [41] suggest researchers consider “*how far they are willing to go*” in persuading policymakers to adopt research evidence – “[W]*hen does persuasion tip into cynical manipulation?*” [41] They say that that “*successful engagement in ‘evidence-based policymaking’ requires pragmatism, combining scientific evidence with governance principles and persuasion to translate complex evidence into simple stories*” [41].

### Contextual research

To create baseline data for evaluation of KTE strategies, which can also inform site-specific research priorities, it is necessary to understand the levels of stakeholder knowledge, skills, attitudes and actual practice in regard to PTB and its prevention. The contextual research we propose informs our understanding of current knowledge and practice and the prioritisation of site-specific KTE research. It provides invaluable baseline information for evaluation of KTE strategies eventually implemented at these sites. Stakeholder engagement in this research will help to ensure the relevance, applicability and sustainability of strategies that emerge.

- The overarching aim for all stakeholder groups in each Preterm Birth Initiative setting is to learn more about how these stakeholders understand PTB, its determinants, complications and solutions, as well as to learn whether current prevention and care practice in these settings is evidence based. It is also important to learn about barriers and facilitators to stakeholder uptake of PTB knowledge in their respective contexts.
- For individuals, families and communities, we need to understand what each of these groups considers to be the most important PTB outcomes and the within group variability in prioritisation, as well as to know their views on optimal ways to facilitate change in government and other health policies that will improve PTB outcomes.
- For providers, it is important to know whether they are aware of efficacious interventions for preventing PTB and caring for mothers and infants affected by PTB. In each setting, we need to understand provider knowledge, skills and attitudes to implement and promote KTE strategies to reduce PTB among mothers, families and communities. We also need to know what barriers exist to implementation of such KTE strategies.
- For policymakers, it will be crucial to know the degree to which PTB is prioritised in the health agenda. If PTB is not considered a pressing issue, we should try to learn what barriers exist to prioritising it, and then work with policymakers to overcome these barriers.

An additional contextualising question in each stakeholder group is to understand current practice for advancing KTE for PTB. We will want to help them refine and adapt evidence-based KTE strategies for implementation and evaluation in their respective contexts and settings. Some questions we may ask might include, What are the KTE approaches currently in use across multiple sectors (e.g. MNCH, primary care, community/public health)? How is the impact of these strategies being evaluated?

### KTE strategy research

To have a population-level impact on the burden of PTB, comparative research is necessary to determine the relative effectiveness of different KTE strategies as applied specifically to PTB, including combinations or ‘bundles’ of KTE strategies. Bundles, in this case, reflect the fact that specific KTE research has taken place, although traditionally more so with providers and far less with consumers or policymakers. The field may thus be ready to implement combinations of strategies shown to be effective singularly, but never tested in combination. There is substantial room for testing effectiveness of differing sets of bundled interventions respectively appropriate to each of the three different audiences.

Limited systematic review evidence is available on optimal KTE strategies specific to PTB. Much of the evidence is derived from reviews of KTE strategies used in other aspects of MNCH, as well as from reviews cutting across many fields of health and medicine. Programmatic ‘grey literature’ (i.e. reports, summaries and analyses published by governments, non-governmental organisations, universities and other kinds of organisations) could provide some PTB-specific or at least MNCH-specific evidence, but almost none of this literature has been reviewed systematically. Given the relatively recent understanding regarding the importance of focusing on KTE strategies to maximise effective dissemination and diffusion of evidence, many additional studies are needed, each linked to a different audience potentially invested in implementing evidence-based practices. Recognising the variability within and among audiences, and that some may be less or more open to implementing change, the following types of KTE research are needed:

- An overarching aim is to investigate the best ways to engage in KTE across all audiences – communities, families, front-line health workers, healthcare systems personnel and policymakers. We need to understand how they might best collaborate in KTE around PTB. We need to measure population-level impact if we are to reduce the burden of PTB. Another aim is to understand whether this type of landscape assessment should occur concurrently or sequentially. For example, is it less or more important to begin at the individual versus the policymaker level, or are efforts needed at all levels of audience simultaneously?
- For individuals, families and communities, we need to investigate which KTE strategies increase the likelihood of their accessing, understanding, adopting, and maintaining evidence-based PTB practices with reasonable fidelity.
- For providers, we need to determine the best ways to train them in KTE strategies that improve provider practice and facilitate outreach to community and policymakers.
- For policymakers, we need to know more about which KTE strategies increase the likelihood that local or national health policies will be informed by systematic review evidence.

### Evaluation

To build evidence on which KTE strategies can effectively be promoted and sustained, we need to know which KTE strategies are effective and can be sustained to increase the impact of PTB interventions.

- For individuals, families and communities, we need to compare pre- and post-intervention impact of KTE strategies in terms of (1) access to systematic review evidence on PTB among the community and providers; (2) changes in individual and community beliefs and norms about the cultural relevance and effectiveness of KTE; (3) repeated, long-term data comparisons of programme effectiveness; and (4) overall changes in PTB outcomes.
- For providers, we need to compare pre- and post-intervention impact of KTE strategies on (1) access to systematic review evidence on PTB and KTE; (2) adoption of systematic review evidence into new practice; (3) repeated, long-term comparisons of KTE strategies; and (4) overall changes in PTB outcomes.
- For policymakers, we need to compare the pre- and post-intervention impact of KTE strategies on (1) access to systematic review evidence on PTB and KTE; (2) new policies informed by systematic review evidence; (3) repeated, long-term comparisons of KTE strategies; and (4) overall changes in PTB outcomes.

### Infrastructure

A precondition for effective KTE is a communications platform by which up-to-date scientific knowledge can be shared with people in positions to make a difference in PTB outcomes. While we found no existing platforms devoted to the transfer of knowledge on preventing and treating PTB, we identified several effective models from other health-related fields that could inform the Preterm Birth Initiative’s efforts. A high priority is to create such platforms for policymakers, providers and the populations most affected by PTB. We recommend multiple (possibly interconnected) communication platforms targeting all stakeholders. Some examples of these platforms could include the following:

- A science-based PTB public outreach campaign that would ‘push out’ information to those individuals and families most at risk for adverse PTB health outcomes. Public education and outreach initiatives are widely recommended, and are effective at increasing consumer access, understanding and adoption of behaviours that promote health [40, 42]. Those using digital and social media platforms will have the widest reach. While knowledge is insufficient for sustained change, it is an important cornerstone of interventions aimed at achieving behavioural change.
- A PTB health evidence web portal for providers would offer a clearinghouse for evidence on the effectiveness of prevention and treatments for PTB. Such a service would be organised around a website that would provide its users access to summaries of quality-rated systematic reviews, which could be searched by topic (e.g. premature birth, maternal depression, etc.). The site would make accessible additional resources for provider trainings, service-based learning programmes and team-based learning. Video clips and curriculum materials suitable for use in classroom trainings, distance-learning programmes and tools for self-assessment would also be part of this resource. Furthermore, parallel materials aimed at reaching consumers of varying educational and reading levels would also be made available on this site.
- A ‘one-stop shop’ on PTB for policymakers that would include optimally packaged online systematic review products, key data and evidence briefs (short, accessible summaries of systematic review and local evidence, describing the context, problem and policy options, and paying attention to issues such as policy implementation, equity, local applicability and the quality of the underlying evidence). Additional capacities might include deliberative dialogues based on evidence briefs (these are in-person discussions between researchers and policymakers, typically followed by a year-long service in which policymakers receive evidence updates). Finally, rapid-response units would provide written summaries and telephone or in-person consultations about best evidence to policymakers and health system leaders. These teams would be flexible and responsive to the evidence query from policymakers as they deliberate prioritisation and resource allocation.

## Conclusions

The opportunity to bring the strongest available evidence to bear in developing the next generation of programmatic efforts aimed at reducing PTB is an exciting and compelling endeavour. To accomplish this vision, a combination of implementing and scaling up use of the best research evidence available will need to be combined with strategies that have been shown to work in KTE. Based on an extensive review of the literature, continuing the usual approach of implementing research knowledge in a somewhat arrogant, ‘cookie-cutter’, ‘top-down’ manner would likely continue to result in unsustainable and unsatisfactory impacts on PTB. Given past history, this top-down approach would provide limited benefit, at best, to communities affected by PTB, including individual women and their infants, partners and other family members, providers, policymakers and other kinds of collaborators. Millions of infants would continue unnecessarily to die or to live with significant avertable health challenges. Instead, the KTE Working Group proposes a holistic view of how health research evidence relevant to PTB, and knowledge of real conditions, in programmatic settings for the Preterm Birth Initiative may be communicated and exchanged among important stakeholders. These stakeholders include UCSF researchers and implementers and, with the ‘one-stop shop’ and the eventual stream of publications, even key stakeholders beyond the Preterm Birth Initiative.

The KTE Working Group presents a range of efficacious KTE strategies as well as a nuanced framework for considering the use of these strategies. We have also noted whether or not they have been tested in MNCH contexts. Use of locally applicable and culturally acceptable KTE strategies in implementing effective and locally applicable interventions will help the Preterm Birth Initiative meet its goals, whilst simultaneously advancing KTE research in general. As we have described, specific strategies are best brought into play with specific kinds of audiences – ranging from the woman herself and her community, providers committed to improving birth outcomes and policymakers, all of whom may be faced with making difficult decisions with few resources at hand. Without effective KTE strategies, and the opportunity to test promising new KTE approaches, the Preterm Birth Initiative’s vision will remain largely unfulfilled. There also remains a significant body of KTE research questions that must be tested to learn whether new combinations of strategies (for example, bundled KTE services) will promote the use of systematic review evidence in healthcare decision-making among all stakeholders in PTB.

Finally, if we are truly to engage in a knowledge ecosystem, founded on humility, we must attend carefully to the ‘E’ in KTE. We must value and promote a true and open ‘exchange’ of knowledge around PTB, and seek a rich understanding of the real conditions and complex contexts in which PTB stakeholders make life-changing decisions. Our task is to optimise not only the ways in which we communicate with stakeholder groups about research evidence, but also to understand what really matters to them about PTB and which problems and possible solutions are applicable to their own lives. Additionally, we need to better our understanding of existing cultural, infrastructural and political settings. The Preterm Birth Initiative will succeed if we approach stakeholders with humility, as equal partners in a goal of improving their very local rates of infants being born at full-term, and improving the lives of infants who are born early.

## Additional file

Additional file 1: Methods for searching the knowledge transfer and exchange research literature. (DOCX 15 kb)

## Abbreviations

KT: knowledge transfer (or ‘knowledge translation’)
KTE: knowledge transfer and exchange
Linking RTA: Linking Research to Action
MNCH: maternal, neonatal and child health
PTB: preterm birth
SUPPORT: Supporting Policy-Relevant Reviews and Trials
UCSF: University of California, San Francisco.
