# Supplementary material for: Preterm birth: the role of knowledge transfer and exchange
Source: Health Res Policy Syst. 2017 Sep 6;15:78. doi: 10.1186/s12961-017-0238-0 (PMC5586007; doi:10.1186/s12961-017-0238-0)
Supplement: Additional file 1: — Methods for searching the knowledge transfer and exchange research literature. (DOCX 15 kb) [file 12961_2017_238_MOESM1_ESM.docx]

**Additional file 1**

**Methods for searching the KTE research literature**

One author (HH) conducted searches of the scientific literature on August 31, 2014 in an effort to identify systematic and narrative reviews of efficacious KTE strategies, and ideally “systematic reviews of systematic reviews” describing such strategies.

Using a range of relevant terms, we searched titles, abstracts and keywords in PubMed and the Cochrane Database of Systematic Reviews. The latter resource includes the Database of Abstracts of Reviews of Effectiveness (DARE). We also searched the Health Systems Evidence database at McMaster University.

Search terms included the following, in appropriate combinations:

- Systematic review OR review OR Cochrane Database of Systematic Reviews
- (Knowledge OR information) AND (transfer* OR translat* OR uptake OR action OR integrat* OR implement* OR disseminat*)
- Preterm birth OR prematurity OR “maternal health” OR “maternal and child health” OR (mother* and child*) OR “women’s health” OR “child health” OR pregnancy OR neonat* OR (clinical AND practice) OR (health* AND policy) OR (lay* OR consumer*)

One author (HH) screened search results (k= 775) and identified reviews for inclusion (k=19).

The identified reviews fell generally into two categories:

1. Reviews of KTE strategies, irrespective of the health care intervention context in which they had been applied (“Cross-cutting KTE”), k=7; and
2. Reviews of KTE strategies applied specifically to maternal, neonatal and child health (MNCH) interventions (“MNCH KTE”), k=12.

Cross-cutting KTE reviews considered the efficacy of specific KTE strategies in any area of health care – for example, “decision aids” for people facing health treatment or screening decisions. MNCH KTE reviews considered the effectiveness of specific KTE strategies in health care contexts specifically relevant to the Preterm Birth Initiative – for example, “decision aids” to improve care for pregnant women.

One author (HH) extracted from each included review its citation information and scope, key characteristics of included primary studies (or included reviews) and its key findings. We synthesized evidence from these findings primarily in tabular form, organized by stakeholder type.
